# The struggle of translating science into action: Foundational concepts of implementation science

**DOI:** 10.1111/jep.12741

**Published:** 2017-03-31

**Authors:** Frances Rapport, Robyn Clay‐Williams, Kate Churruca, Patti Shih, Anne Hogden, Jeffrey Braithwaite

**Affiliations:** ^1^ Centre for Healthcare Resilience and Implementation Science, Australian Institute of Health Innovation Macquarie University Australia

**Keywords:** evidence‐based health care and policy, health services research, implementable research findings, implementation science

## Abstract

**Rationale, aims, and objectives:**

“Implementation science,” the scientific study of methods translating research findings into practical, useful outcomes, is contested and complex, with unpredictable use of results from routine clinical practice and different levels of continuing assessment of implementable interventions. The authors aim to reveal how implementation science is presented and understood in health services research contexts and clarify the foundational concepts: diffusion, dissemination, implementation, adoption, and sustainability, to progress knowledge in the field.

**Method:**

Implementation science models, theories, and frameworks are critiqued, and their value for laying the groundwork from which to implement a study's findings is emphasised. The paper highlights the challenges of turning research findings into practical outcomes that can be successfully implemented and the need for support from change agents, to ensure improvements to health care provision, health systems, and policy. The paper examines how researchers create implementation plans and what needs to be considered for study outputs to lead to sustainable interventions. This aspect needs clear planning, underpinned by appropriate theoretical paradigms that rigorously respond to a study's aims and objectives.

**Conclusion:**

Researchers might benefit from a return to first principles in implementation science, whereby applications that result from research endeavours are both effective and readily disseminated and where interventions can be supported by appropriate health care personnel. These should be people specifically identified to promote change in service organisation, delivery, and policy that can be systematically evaluated over time, to ensure high‐quality, long‐term improvements to patients' health.

## 1 BACKGROUND

This article concentrates on how implementation science can be progressed most effectively and how it is currently perceived within the health services research arena. Clarification of the pathways to implementation of interventions and new knowledge is necessary if we wish to improve care, and ensure policies surrounding health care organisation and service delivery are well grounded. This article presents critical thinking that centres on (1) foundational concepts in implementation science; (2) models, theories, and frameworks for advancing implementation science; and (3) what is persistently interfering with the smooth implementation of research evidence into practice. We offer suggestions for researchers to consider when planning a research study regarding what might improve the impact of their study findings through stronger implementation plans. As a contested and complex topic, with unpredictable uptake of research findings into routine clinical practice, and different levels of assessment and evaluation, it is vital that we take the time to visit the basic precepts of implementation science and the theoretical propositions underlying the production of strong research outcomes. We hope to provide researchers with a yardstick against which to consider whether their research has clear and extensive scope. If we can get it right, this can support the design of new interventions into health care services to bring about longer‐lasting impact and better value‐for‐money, in spite of fluctuations in service delivery, system organisation, and economic growth and decline.

## 2 FOUNDATIONAL THEORETICAL CONCEPTS IN IMPLEMENTATION SCIENCE

Implementation science, dissemination and implementation (D&I),^1^ evidence‐based interventional dissemination,^2^ and implementation research,^3^ is a basket of terms that refers to the application of effective and evidence‐based interventions, in targeted settings, to improve the health and well‐being of specific population groups.^4^ Implementation science is the scientific study of methods that take findings into practice, while “effective implementation” refers to the process whereby an actionable plan is appropriately and successfully executed. Both the science and the implementation elements inspire new knowledge production and its dissemination.^5^ Implementation science enables questions to be asked about whether, and if so how, an intervention can make a difference to a patient's life or to the practice of a health care delivery team, and whether bringing new knowledge into one setting automatically, or with effort, enables its applicability in another. Answers to these kinds of questions can encourage better, more targeted service provision and policy development, and help to remind us of the need to foreground patient care and its delivery with rigorous evidence.

The literature suggests that evidence‐based interventions must be appropriately disseminated (knowledge translation), to the right audiences (knowledge targeting), implemented at the right time (knowledge fidelity); and following dissemination, successfully adopted (knowledge take‐up) and evaluated (knowledge assessment), to clarify the extent to which they are effective (knowledge results).^6^, ^7^ When this works well, outcomes can affect both policy and practice, and can be measured in terms of their long‐term sustainability and translational effect (knowledge evaluation).^8^ However, there are multiple challenges to effective dissemination in health services research, not least the ability to communicate and move research findings beyond the scope of an immediate project to influence health care delivery systems and procedures, and long‐term policy initiatives and sustained, ubiquitous practice change (knowledge spread). Other challenges include how evidence can best be defined, the context within which evidence may be successfully implemented, and how to keep evidence relevant within rapidly adaptive, changing health care systems.^8^, ^9^, ^10^

With this in mind, in 2008, Rabin et al recommended returning to first principles in implementation science, to clarify the evidence‐base of an intervention. We would argue that the same applies today, almost a decade later. We need to remind ourselves of implementation science's foundational concepts and develop research strategies according to these concepts. We need to discuss their incorporation right at the outset of a rigorous health services research design, recognising that this will help us to understand more about the form that new knowledge takes, and how to bring new knowledge to the awareness of others. In describing the foundational concepts of implementation science in the section that follows, we wish to highlight that the foundational concepts are critical elements of a successful implementation process.

**Figure 1:**
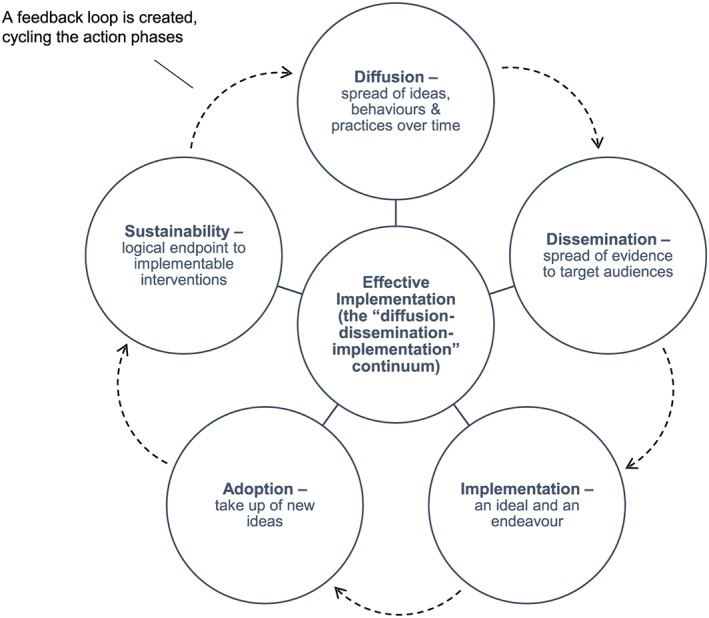
Foundation concepts: the 5 categories of implementation science

Foundational concepts, which underpin an intervention's proven efficacy and effectiveness, are said to fall within 5 distinct categories: (1) diffusion, (2) dissemination, (3) implementation, (4) adoption, and (5) sustainability^1^ (see Figure 1 for the authors' visualisation of Rabin's foundational concepts). There are slight variations to this schema, most notably, the categorisation provided in Roger's “implementation science model,”^7^ which includes “evaluation” and “institutionalization.” We would also emphasise the point, as other scholars of implementation science have, that context is crucial to successful adoption, take‐up, and spread.^11^, ^12^, ^13^ Additionally, as Stetler et al^11^ and Øvretveit^13^ remind us, we must not only consider the different environments where we want to ensure implementation can take place but also that context and environment influences much that is important. However, if we take for the moment the 5 categories Rabin et al^1^ presented, we can consider them as a comprehensive suite of core components that together support an intervention. Each has a powerful message to deliver, and together can act concertedly to influence the development of implementation support tools such as guidelines, programmes, projects, and policies.

“Diffusion,” while loosely defined in many studies, is the notion that ideas, behaviours, and practices spread out in a relatively unfocused way, through informal and formal communicative channels, over time.^6^, ^7^ Most experts see diffusion as relatively passive, where little targeted planning takes place. In effect, diffusion, as a foundational concept, is part of “a diffusion‐dissemination‐implementation continuum”^1^ and as a result, whether researchers are directly involved in how behaviours and practices are dispersed through a system or not, diffusion, when it occurs, is to a considerable extent, emergent and spontaneous.^14^

“Dissemination” is “an active approach to spreading evidence‐based interventions to a target audience via determined channels using planned strategies.”^1^ The “planned” and “targeted” components of dissemination are often underpinned by strategic thinking about how to reach as wide an audience as possible, efficiently and effectively, and in the least possible time, while leaving the basic structures and processes that make dissemination feasible undisturbed.^15^

“Implementation” is both “an ideal and an endeavour.”^8^ For an ideal, it captures research evidence and applies it to practice, reaching out, through science or social science, with a message about systems‐ or organisation‐based levels of change. As a practice, translating research into implementable procedures “recognizes that these stages do not happen automatically, often to no great extent, and sometimes not at all.”^8^ When implementation fails, it can leave behind a legacy not just of wasted economic resources but also at worst, of systems upheaval, affecting health professionals at various levels, and impacting on patient care. When genuine implementation is successful, it can affect whole systems or services, positively improving practice and optimizing patient care. We have presented a graphic representation of the difference between the attempt to turn research into implementable procedures (the implementation stage; ideal and endeavour) and the genuinely successful implementation of an intervention (the effective implementation stage; the successful endpoint) in Figure 1. In effective implementation, all the component parts come together to impact on the system or service; in effect, this is the central focus around which all other component parts circle. When unsuccessful, challenges to the service or individuals must be addressed quickly, but this can be delayed as a result of the complexities of health care service organisation and delivery, often leaving patients' needs unmet.^8^

“Adoption” is the degree of uptake of new ideas, behaviours, practices, and organisational structures. As we have seen, adoption is dependent on the context within which implementation of an intervention or diffusion or both have taken place, and in turn, the context is influenced by the practices and attitudes of those working within a particular organisation, the experiences of those presiding over organisational design and activity, and organisational structure and processes.^14^, ^26^ Also at play are the resources at the disposal of those wishing to mobilise activity. These include the material resources, the aptitudes of staff, and “the policies and incentives, networks, and linkages”^14^, ^26^ that affect how information is used by adopters.

“Sustainability” is the logical endpoint of implementable interventions, once new knowledge and the intervention have been successfully applied and embedded. That an intervention, once implemented, should be sustainable is said to create “a feedback loop that cycles through the action phases” of an intervention^16^, ^21^; a feedback loop that demands monitoring, adoption, and extended uptake phases, so that with each cycle, the intervention becomes more firmly entrenched within a system. However, for something to be sustainable, it is not enough to just measure the success of its evidence base. It also needs to take account of the real‐world environment setting.^2^ This aspect of sustainability cannot be underestimated. Recognising the demands of different health care environments and their complexities adds to our understanding of the resources necessary to sustain uptake and the initial commitment necessary for people to get involved in the first place.^17^ We can bring this together in a conceptual model. Figure 1 depicts the interrelationships between the core concepts.

It is important to emphasise that, as outlined in Figure 1, these components act connectedly and are integrated and should not be perceived as acting interdependently of one another. To make this point, in Figure 1, we present them in a circle, to give each an equal weight and position and to reinforce the point that they are bound to one another as cohesive elements of 1 effective implementation, rather than as discrete elements within a pecking order or hierarchy. It is also worth noting that the directionality indicated in Figure 1, where 1 component leads on to the next, can change. For example, adoption may come before implementation, not after, if the intention to implement is dependent on the success of the adoption of an intervention. Ordering of components is dependent on circumstances, settings and situations.

## 3 THEORIES, FRAMEWORKS, AND MODELS FOR ADVANCING IMPLEMENTATION SCIENCE

Building on these points, theories, frameworks, and models has been created to explain or advance implementation science. These are designed to (1) support the successful spread of D&I, (2) clarify what influences research outcomes, and (3) help evaluate the success of the intervention.^18^ However, theories, frameworks, and models are always simplifications of the messy real world. Concepts within theories, frameworks, or models, so critical for underpinning the way an intervention is appropriately implemented, or supporting new knowledge getting embedded into practice, can often go unnoticed in favour of rushing towards the actualisation of the implementation process itself and sustaining interventional viability. As a result, theories, frameworks, and models are infrequently incorporated into health services research studies at the research design stage where they are needed most, to legitimise the work planned, and yet without them, these kinds of study findings may be less meaningful, and their resultant legacies more short lived.^19^ Eccles et al^20^ noted that “uptake of research findings into routine health care is a haphazard and unpredictable process,” emphasising that “the usefulness of the results of implementation studies is limited, due in part to the lack of any underlying framework of the important dimensions of research studies in this area and the health care settings within which they are conducted and may subsequently be used.”^20^ In 2009, Eccles et al^18^, ^19^ presented us with an implementation research agenda. It aimed to emphasise that, as part of a battery of considerations for implementation research, frameworks are “potentially useful tools for considering the issues that a research agenda needs to address.” Eccles et al^19^ also commented on the use of theories, highlighting that the benefits they offer are 3‐fold: (1) “a generalizable framework that can apply across different settings and individuals”; (2) the possibility of “incremental accumulation of knowledge”; and (3) a way of framing analysis work. While these suggestions were clearly set out in their publication and reiterated again, as part of the High Level Group on Clinical Effectiveness's manifesto,^21^ we reflect that there is a clear need to return to the points they made as uptake has since proved limited, and by so doing, we wish to emphasise the value of an ongoing consideration of each term and a clearer understanding of the role they play in implementation science (see section below for clarification).

### Box 1: Defining theories, frameworks, and models

**Figure 2:**
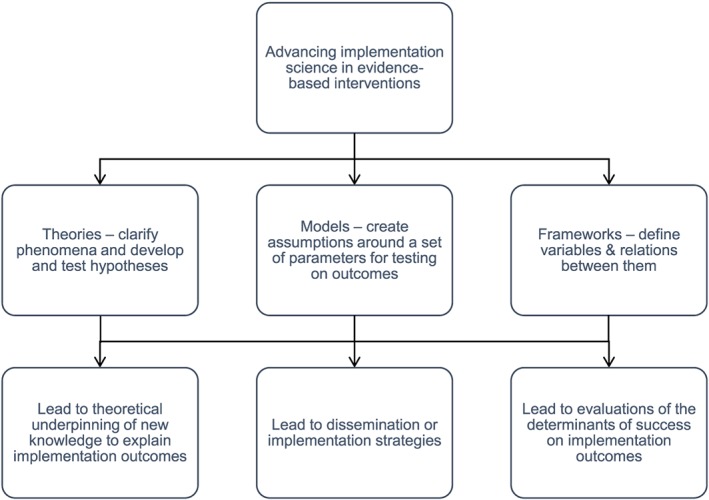
Theories, frameworks, and models: supporting the successful spread of dissemination and implementable study outcomes

The difference between theories, frameworks, and models may need some unpicking (see Figure 2), if we wish to understand their ability to move implementation science forward. “Models,” according to Carpiano and Daley,^22^ have a narrower focus than theories and frameworks. Models are designed to enable specific assumptions to be made about a set of parameters or variables that can then be tested on outcomes, using predefined methods. “Frameworks,” on the other hand, define more broadly “a set of variables and the relations among them,” while “theories,” nested within conceptual frameworks, enable researchers to make assumptions that help clarify phenomena or develop and test hypotheses.^22^, ^p.565^

While we may accept theories, frameworks, and models as distinct concepts, we recognise that in health services research, they are often unhelpfully conflated.^18^, ^22^ Meyers et al^23^ have suggested that this results from lack of standardized language to describe and assess implementation. As separate entities, the section that follows aims to highlight their different qualities and strengths, by concentrating on 3 very different uses and applications of models, theories, and frameworks. Each of the following examples has been purposefully chosen to emphasise their distinctive nature: (1) models for advancing the implementation of evidence‐based interventions; (2) implementation of new knowledge according to “social cognitive theory (SCT)”; and (3) implementation frameworks for evaluating success.

### 3.1 Models for advancing the implementation of evidence‐based interventions

In 2012, Tabak et al^24^ undertook a detailed review of models specifically used in D&I research and published in peer‐reviewed journals. Focusing on 61 models, derived across disciplines such as Health Services Research, Nursing, Public Health, and Medical Science, including models presenting “innovation, organizational behaviour, and research utilization,” Tabak et al^24^ categorised each model according to construct flexibility, dissemination or implementation angles, and a socio‐ecological framework. In listing all 61 models, Tabak et al^24^ hoped to raise awareness of the diverse range and scope of these models, but in effect, highlighted their extensive degree of overlap, arguing that there was some scope for having a model in place in 1 discipline that could be used in another. They also identified the value of models for a wider understanding of implementation constructs. This final use of models, defined by Nilsen^3^, ^18^ as “process models,” is said to be suitable for describing or guiding “the process of translating research into practice.”

Of the 61 models of Tabak et al,^24^ most were already in existence, used extensively in 1 discipline, and presented exactly as they had been designed, while others were adapted for use for different applications or contexts, or adopted across different disciplines. In the latter case, the use of a preexisting model was said to be highly beneficial and supportive of the notion that a model has generalizability and multiple uses. Adaptation of a previously adopted model to suit a new study's needs was also said to develop confidence in the model's prevalidated measures and allowed for further testing and assessment. The suite of models of Tabak et al^24^ included those supporting transfer, dissemination, and improvement, such as a “‘Model for Locally Based Research Transfer Development’ and a ‘Model for Improving the Dissemination of Nursing Research.’” The models predominantly covered either dissemination or implementation strategies but not generally both and spanned an extensive and varied topic base, such as health promotion, improving health services research dissemination, coordinating implementation, policy process, knowledge infrastructure, social marketing, patient safety, technology transfer, and evidence‐based practice in public services.

For Tabak et al,^24^ it was not only the model that was important but also whether there were measures in place to define and assess the model's constructs, so that it could be rigorously operationalized. Many of the models did not include construct measurements; however, indicating that standard measurement development is still somewhat in its infancy. Without greater awareness of reliable and valid measures, an assessment of common constructs cannot take place, resulting in, for example, shortcomings in information about outcomes and units of analyses.^24^

### 3.2 Implementing new knowledge in health services research: the example of SCT

Some theories have been specifically developed to underpin new knowledge and its take‐up in practice, through implementation science. These are often known as “research‐to‐practice” theories.^7^, ^18^ For example, SCT was developed as a broad‐based conceptualisation for understanding clinical behaviour change. It is widely used in implementation science, originated in psychology, but is now applied in other fields, to explain implementation outcomes.^18^ Social cognitive theory is helpful for understanding the determinants of clinical behaviour and can support a clearer picture of “cognitive processes involved in clinical decision‐making and implementing EBP (evidence‐based practice).”^7^, ^18^ While originally designed to examine behaviour‐change at an individual level, SCT is now also used for its applications to wider groups of health care professionals' to examine aspects of the efficacy and effectiveness of practice.

In 2008, Godin et al^25^ undertook a systematic review of behaviour change studies and factors that influenced health care professionals' behaviour using SCTs, the first systematic review of its kind for clinically related behaviours. They identified 72 studies that provided information on the determinants of intention, and 16 on the determinants of behaviour, referencing, in particular, “*The Theory of Reasoned Action*,” and “*The Theory of Planned Behaviour*.” They argued that prediction of behaviour and action was predicated not only on determinants of intention (such as role beliefs and moral norms) but also on the type of health care professional involved in the research, individual characteristics of professionals involved, social influences impacting on their involvement, and methodologically: eg, context, sample size, efficacy, and objective/subjective measures. Since this work, Cane et al^26^ have presented an integrative theoretical framework for behaviour change research and tested its validity for cross‐disciplinary implementation. Their “Theoretical Domains Framework” has been well tested across health care systems and aims to bring about positive behaviour change.

### 3.3 Determinant frameworks for evaluating success

Determinant frameworks have been cited as 1 way of evaluating the variables of success and are valuable in understanding the influences on implementation outcomes.^18^ They are not theories and cannot clarify how change has taken place. However, they can be used to explain the outcomes of implementations, such as behaviour change in health care professionals or professional adherence to, and uptake of, clinical guidelines. Determinant frameworks are useful in defining both dependent and independent variables influencing implementation outcomes. They can draw links between dependent variables and can highlight barriers between variables that hinder interdependence and therefore have an impact on implementation. They can bring to others' awareness the strengths and weaknesses of implementable outcomes and can assist with the design and execution of implementation strategies regarding, for example, changes to clinical guidelines. Meyers et al^23^ undertook a synthesis of the critical steps in the implementation process, including postimplementation steps such as those necessary to realise determinants of success. They undertook a detailed review of implementation frameworks (based on empirical, theoretical, and conceptual work), detailing 14 specific aspects, phases, or “steps” to the implementation process, derived from an assessment of 25 frameworks.^23^ The review related to the implementation of evidence‐based programmes across literature sources target populations and innovation types. Their results emphasised the need for clear implementation structures built in to a study from the outset. In relation to postimplementation assessment, implementation frameworks were described in terms of the need for *“Ongoing Structure Once Implementation Begins”* and to improve future applications (italics and capitals in original).^23^ Meyers et al^23^ defined the final phases of implementation frameworks as important for clarifying (1) that progress had been made in the implementation for the benefit of all stakeholders involved; (2) a retrospective analysis of implementation had been undertaken; (3) assessment of strengths and weaknesses embedded in the process was underway; (4) testing and modification of the implementation was possible, according to (5) self‐reflection and critical awareness, and the reconceptualisation of what quality implementation should look like. Meyers et al^23^ highlighted that between frameworks, there was substantial agreement about the steps and phases necessary for successful implementation and evaluation (the determinants of success), with framework developers valuing

“Monitoring implementation […] developing buy‐in and a supportive organizational climate […] technical assistance […] feedback mechanisms […] the creation of implementation teams […] and the importance of building organizational capacity.”^23^, ^p.471^

Their work emphasised the importance of ongoing monitoring and evaluation for positive impact of the implementation process and resulting outcomes. Having covered core ideas about theories, models, and frameworks, we turn to considering aspects of translation.

### 3.4 Translating health services research findings into practice

We have emphasised the importance of health services researchers ensuring that appropriate theories, models, and frameworks are in place to give support to their implementable study outcomes. We have discussed the value of returning to first principles, to define the core concepts of implementation science and clarify an intervention's evidence base.^1^ Yet even with all of this in place, sustaining positive change to systems, processes, and behaviours seems difficult to achieve, while new policy or practice initiatives within the health services do not always withstand the test of time.^27^ We have described elsewhere how challenging it is to plan for the translation of knowledge into practice, particularly around transitional care programmes.^28^ In the final sections of this article, we would like to raise a selection of the most pressing problems health services researchers may face, highlighting what may be getting in the way of progressing long‐term implementation plans before considering how these may be alleviated and where we go from here.

## 4 LANGUAGE AND TRANSFORMATION

Challenges surrounding implementation, and creating a science of implementation in health services, are particularly noticeable when it comes to clarifying shared understandings of the terms used to describe the implementation process.^28^ This is exacerbated by a lack of transformative vision and planning that would allow health services researchers to achieve their research aspirations. Grol and Grimshaw^29^ have alluded to this when they say: “sometimes the step from best evidence to best practice is simple; however, most of the time it is not, and we need various strategies targeting obstacles to change at different levels.” To achieve long‐lasting change in the delivery of health services to patients, according to appropriate care delivery models, systems, and practices, we need a common language. A language that is clear enough to withstand knowledge translation in all its variation and to overcome semantic nuance, which often gets in the way of implementation, leading to misunderstandings around what people are attempting to do and hoping to achieve.

Terminology can drive a wedge between researchers and service providers, researchers and service users, and researchers and policy developers, who need to understand one another to bring about wider service reorganisation and practice improvements. When there is semantic disjuncture, miscommunication is rife. This is not helped by the fact that health services researchers see implementation science in different ways. For some, it is the synthesis of evidence into practice. Others concern themselves with integrating medical advances into trials, and still others define it as the preemptive strategies for ensuring the translation of knowledge, such as (1) undertaking systematic literature reviews; (2) designing new technologies; and (3) changing infrastructure support.^5^, ^29^, ^30^, ^31^, ^32^ Semantic nuance adds a further dimension of complexity to an already tricky issue and can problematize dissemination plans.

We have highlighted earlier that some cornerstone concepts of implementation science are defined by very specific terms with which researchers need to familiarise themselves, if they wish to design a rigorous study: (1) diffusion, (2) dissemination, (3) implementation, (4) adoption, and (5) sustainability. Yet how many of these terms are clearly understood by those stakeholder groups who will be called upon to play an integral role in the implementation process, and without that clarity, how can their value as part of that process be best communicated? The language of implementation science needs to be consistent yet accessible to all. For this to be successfully achieved, researchers must err on the side of overexplanation, forming close ties with all stakeholder groups so that language can be widely adopted and consistently understood. The language of implementation science must be accessible and communicative, to benefit not only other researchers but also practitioners and other stakeholder groups involved in the implementation process.

**Figure 3:**
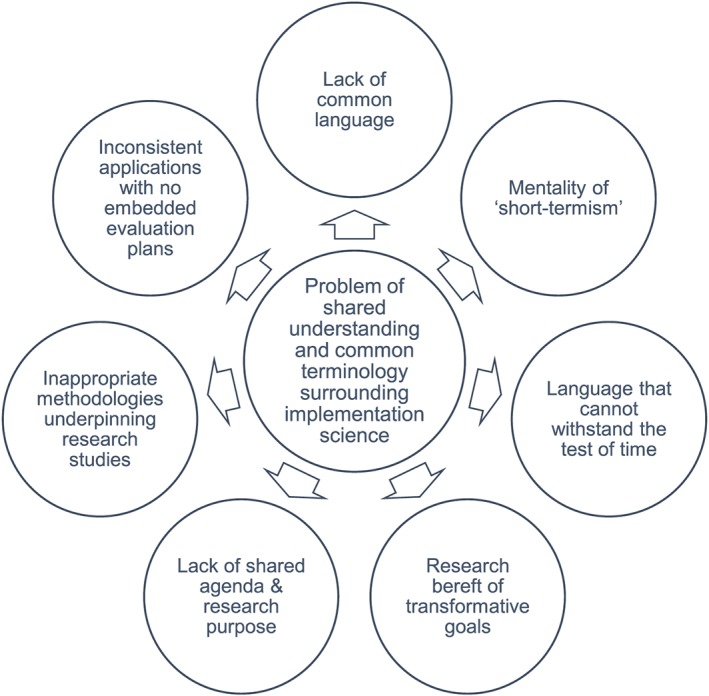
Challenges of implementation science: language, methodology, and transformative vision

Finally, health services research designs should have high aspirations to be transformative. Research that is bereft of transformative goals, grounded in the here‐and‐now, and underpinned by inappropriate methodologies can lead to inconsistent outcomes that lack integration into practice and often lack an embedded evaluation plan. We summarise these concerns in Figure 3, suggesting some ways of overcoming or managing these concerns in Box 2.

### Box 2. Suggestions for overcoming some of the challenges of implementation science

**Table:** 

Challenges	Overcoming Implementation Science Challenges
Lack of common language	Common language is essential. Terminology must be consistent and used consistently within and across adopting groups, organisations, and settings. Common language is imperative for the sustainability of an intervention and the clarity about new structures and processes.
Short‐termism	Characteristics of the adopting organisation and adopting community should be not only recognised but also considered in terms of the longer‐term sustainability of the intervention. This includes an organisation's size, complexity, and readiness for change.
Lack of transformative goals	The ability of an adopting organisation or community to share transformative goals will be defined by the attitudes of the adopting organisation. These can be considered in relation to individual and group concerns, individual and group adopters, the ability of an organisation to come on board early or late in the adoption process, and the motivation expressed for implementation to be a success.
Lack of shared agenda	Context and rationale for delivery must be clearly understood and agreed by all constituencies for successful implementation to occur.
Inappropriate methodologies	Fit of methodology to aims and objectives of the implementation process and implementation outcomes must be recognised at the study design stage and underpinned by an evidence‐base.
Lack of embedded evaluation plans	Evaluation frameworks should be in place as part of new research design, ready to be applied at the appropriate stage in the implementation process, for evaluation purposes. Evaluation frameworks provide a clear structure to evaluating implementation endeavours.
Language unable to withstand the test of time	Language must be able to withstand the test of time, thus, if terminology around implementation strategy or evaluation framework is refined, this must be through agreement with all stakeholders and researchers involved, for the common good.

## 5 WHERE DO WE GO FROM HERE?

So where do we go from here? We suggest that it is time for translation endeavours to turn a corner, for health service researchers to contend with a lack of consistency and clarity. Researchers should consider whether they have taken into account how they will obtain evidence about research impact and ensure scientific rigour when they design their research studies.^33^ They should include the user perspective, to inform health care professional opinion,^34^ and create patient‐centred outcome indicators that favour a more thorough outcome‐evaluation nexus.^35^

**Figure 4:**
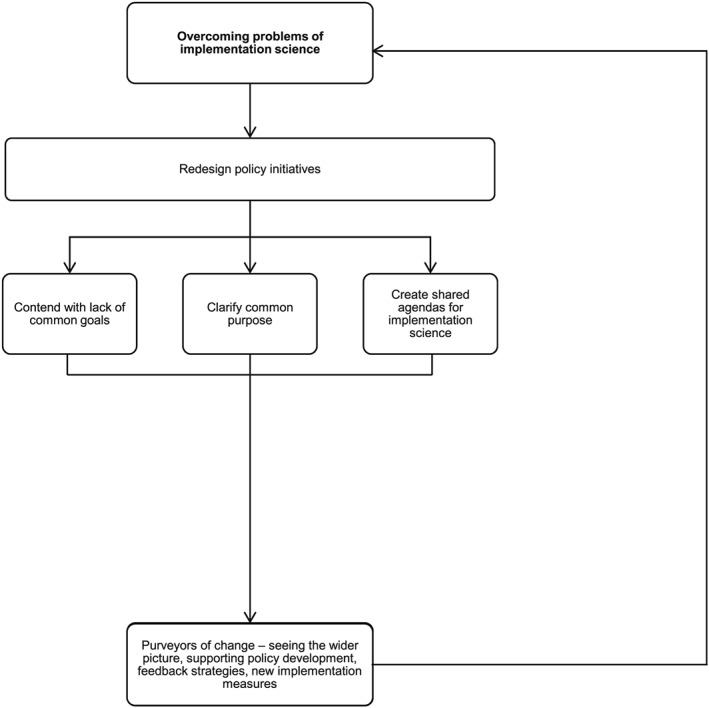
Overcoming some core problems of implementation science

A number of approaches may work concertedly to address the current problems faced in implementing interventions for longer‐term change and to manage more effectively system capacity and scope. Alongside redesigned policy initiatives, contending with common goals and shared agendas, and identifying champions of translational effect may help overcome problems of translational research.^6^ Champions of translational effect (who come in many guises, eg, “boundary spanners”^36^ are people who are recognised for their ability to engage thoroughly with all stakeholder groups, include patients in consultation around implementing study outcomes, and utilise data to serve whole population groups—incorporating a vision for wider‐population impact. Champions of translational effect should be cognizant of a proposal's evidence base and be clear about its intended impact, be aware of strategies to overcome problems that occur along the way to successful implementation, be supportive of indicators that measure success holistically, and be able to manage and monitor progress.^29^ Preparing for change, by having champions of translational effect on board, within the right setting for implementation, and with appropriate resources in place to see implementation through, is critical for success. However, championing translational effect is a process that also needs overseeing, by “change agents.” Change agents are people who can bridge the divide between research outcomes and stakeholder groups.^8^, ^12^ Finally, it is necessary to also identify “purveyors” of change,^37^ who offer support to initiatives through their vision of the wider picture (see Figure 4). Purveyors of change can ensure a programme or practice has integrity and fidelity for the situation or setting within which it is envisaged. Purveyors of change can be instrumental in ensuring that others will persevere with implementation in spite of any problems faced along the way. In effect, they are identified to

- Develop implementation measures that offer stakeholders practical feedback as implementation embeds;
- Influence groups of future purveyors, with the knowledge and skills they have acquired that are necessary to carry implementation forward; and
- Engage policy makers and managers with the notion of successful implementation, looking forwards not backwards, to a future of effective practice, and well‐supported patients.^37^

In the end, researchers may need to consider the value of recommending and supporting the redesign of policy initiatives, with initiatives that are broad enough to manage research and system capacity and scope.^8^, ^33^ Policy initiatives should be in line with patients' need and expectation and developed to overcome the challenge of shared understandings between different stakeholder groups (synthesised in Figure 3). Policy initiatives should also be able to clarify the terminology that will be used, underpinned by the evidence in question, and show that language will be used in such a way that shared understanding is achievable, with goals for all stakeholders and users to aspire to, in the longer‐term as well as the immediate future.

## 6 STRENGTHS AND WEAKNESSES

In this article, we chose to cover extensive ground. We wanted to give an overview of the challenges researchers face when they wish to translate science into action and offer some key concepts in implementation science. By so doing, we recognise that we have had to forfeit the opportunity to go into any great depth on a wide number of critical elements of the subject, not least the models and frameworks that underpin implementation science, which are numerous. We also appreciate that by offering a summation of a complex topic, we have only been able to pay a cursory visit to some of the theoretical frameworks in this field and their applications that, for those interested in pursuing the subject further, are critical to understanding. The broad‐brush approach was an attempt to provide the readership with a greater understanding of the topic as a whole, to elucidate its transformative power, and at the same time, lay the grounding in implementation science's basic principle, and we hope that its contribution will be taken in this light.

## 7 CONCLUSION

Implementation science in health services research has come a long way since, decades ago, the evidence‐based movement took root and laid the ground rules for better science and higher‐quality, patient‐focused care. And yet implementation science is still seen as a minefield. Patients still receive substandard, variable care that is all too frequently inappropriate and unsafe.^38^ Implementation science has some way to go before research outcomes can be achieved that are consistently of a high quality and that impact on health services in such a way that can assure patients their health is in good hands.^27^, ^37^ Until such a time, we urge health services researchers to return to first principles, to lay down the ground rules for their research, discuss rigorous, methodologically sound procedures, and consider how their findings will lead to implementable study outcomes. In effect, researchers need to address the challenges noted in this article to improve health systems and services, meet public and political demands, and drive policies that contribute effectively to future health care developments.

It is highly unlikely that there will be a “quick fix” solution. As Clay‐Williams et al^12^, ^33^ remind us, “changing the culture of health care takes time, clinical areas will adopt changes at varying paces and educational programs will have diverse effects on different groups and services.” Yet if we stick to our guns, clarify language‐use, challenge methodological inconsistency, firm‐up our evidence‐base, and systematically evaluate service integration, we may yet turn implementation science into a cause for widespread benefit, delivering improved patient outcomes.

## CONFLICT OF INTERESTS

The authors confirm that there are no conflicts of interest to declare.
